# Information = equity? How increased access to information can enhance equity and improve health outcomes for pregnant women in Peru

**DOI:** 10.1093/pubmed/fdy177

**Published:** 2018-10-11

**Authors:** Jose E Pérez-Lu, Angela M Bayer, Ruth Iguiñiz-Romero

**Affiliations:** School of Public Health and Administration, Universidad Peruana Cayetano Heredia, Lima, Peru

**Keywords:** electronic health records, health information systems, healthcare disparities, maternal health services, pregnant women, text messaging

## Abstract

**Background:**

The Peruvian health system provides care through numerous, disconnected health establishments and information systems. Our objective was to explore information use and needs of pregnant women to improve quality of care.

**Methods:**

We carried out a mixed methods study in the Lima capital metropolitan area in 15 health centers. This included four focus groups with 34 pregnant women and surveys with 403 pregnant women.

**Results:**

Pregnant women’s information needs depend on their age, number of pregnancies and environment. Women relied on their social networks for pregnancy-related advice and valued high-quality, timely and targeted information from the health system. Participants’ information needs include access to reliable information and responses to their questions in a warm, caring and safe environment. These needs can be met during prenatal check-ups and in group settings through informational talks and visual displays in waiting areas, as well as through appropriate digital technologies such as SMS messages and electronic health records.

**Conclusions:**

Pregnant women need individualized health information in an understandable, secure and friendly manner to maximize their understanding of their pregnancy, follow recommendations and optimize health outcomes. Customizing e-Health programs that reach many pregnant women has greater potential for more equitable health outcomes.

## RESUMEN

**Contexto:**

El sistema de salud peruano brinda atención mediante sistemas de información y centros de salud variados y desconectados. Nuestro objetivo fue analizar el uso de la información y las necesidades de las embarazadas para mejorar la calidad de la atención.

**Métodos:**

Llevamos a cabo un estudio de métodos mixtos en 15 centros de salud del área metropolitana de Lima. Incluyó cuatro grupos focales con 34 embarazadas y encuestas con 403 embarazadas.

**Resultados:**

La información de las necesidades de las embarazadas depende de la edad, la cantidad de embarazos y el entorno. Las mujeres confiaban en sus redes sociales para obtener consejo relacionado con el embarazo y valoraban la información de alta calidad, oportuna y focalizada provista por el sistema de salud. Las necesidades de información de las participantes incluyen el acceso a información y respuestas confiables a sus preguntas, en un entorno cálido, atento y seguro. Estas necesidades pueden suplirse durante los controles prenatales y en entornos de grupos mediante charlas informativas y gráficos exhibidos en las áreas de espera. También por medio de tecnologías digitales adecuadas como ser SMS y registros de salud electrónicos.

**Conclusiones:**

Las embarazadas necesitan información de salud individualizada, en forma entendible, segura y amigable para optimizar su comprensión del embarazo, seguir las recomendaciones y mejorar los resultados de salud. Al personalizar los programas de e-Salud que alcanzan a muchas embarazadas se tiene un mayor potencial para resultados de salud más equitativos.

**Palabras clave:**

Registros de salud electrónicos, sistemas de información sanitaria, desigualdades en la atención de la salud, servicios de salud materna, embarazadas, mensajes de texto

## RÉSUMÉ

**Contexte:**

Le système de santé péruvien fournit des soins par l’entremise de nombreux établissements de santé et systèmes d’information déconnectés. Notre objectif était d’explorer l’utilisation des renseignements et les besoins des femmes enceintes afin d’améliorer la qualité des soins.

**Méthodes:**

Nous avons mené une étude portant sur différentes méthodes dans la région métropolitaine de la capitale, Lima, dans 15 centres de santé. Cette étude comprenait quatre groupes de discussion avec 34 femmes enceintes ainsi que des enquêtes auprès de 403 femmes enceintes.

**Résultats:**

Les besoins des femmes enceintes en matière de renseignements dépendent de leur âge, du nombre de grossesses et de leur environnement. Les femmes s’appuyaient sur leurs réseaux sociaux pour obtenir des conseils liés à la grossesse et valorisaient des renseignements de haute qualité, ponctuelles et ciblées fournis par le système de santé. Les besoins des participants en matière de renseignements comprennent l’accès à une information fiable et aux réponses à leurs questions dans un environnement chaleureux, bienveillant et sécuritaire. Ces besoins peuvent trouver une solution lors des visites prénatales et en groupe grâce à des discussions informatives et des affichages visuels dans les salles d’attente, ainsi que par le moyen de technologies numériques appropriées telles que les messages textes et les dossiers de santé électroniques.

**Conclusions:**

Les femmes enceintes ont besoin de renseignements individualisés en matière de santé de manière compréhensible, sécurisée et conviviale afin d’améliorer la compréhension sur leur grossesse, de suivre les recommandations et d’optimiser les résultats en matière de santé. L’adaptation des programmes de santé en ligne, qui touchent de nombreuses femmes enceintes offre un meilleur potentiel pour obtenir des résultats plus équitables en matière de santé.

**Mots-clés:**

dossiers de santé électroniques, systèmes d’information sur la santé, disparités en matière de soins de santé, services de santé maternelle, femmes enceintes, messagerie texte

## Background

During the last 2 decades, the Peruvian health system has prioritized maternal health care services to reduce maternal mortality, in line with the Millennium and Sustainable Development Goals. While the national maternal mortality ratio decreased from 140 deaths per 100 000 live births in 2000 to 68 per 100 000 in 2015,^1^ disparities in women’s access to quality health services persist. These disparities reflect inequalities in how the health system is designed, which influence how women from different social, cultural and economic backgrounds and regions of the country access quality health services.

The Peruvian health system is fragmented and provides care through numerous, disconnected public and private health establishments. The Ministry of Health (MOH) is the largest provider, covering ~75% of Peru’s population^2^ and providing maternal health care through different types of establishments. Pregnant women can receive prenatal and postnatal care at local health centers (level I) while delivery takes place at specialized maternal centers (levels I–4) or hospitals (level II or III), depending on the complexity of the pregnancy.

This fragmented approach contributes to inequalities among pregnant women and health care providers.^3,4^ For example, women living in isolated rural areas only have close access to level I health centers and need to travel several hours or even days to reach the closest specialized center or hospital. By contrast, those living in peri-urban or urban areas have close access to specialized centers and can receive specialized care throughout pregnancy and delivery. Women’s familiarity with the environment and providers can improve women’s access to ongoing high-quality and clear information and to trustful relationships with providers, reducing their anxieties related to the delivery process. This simple example demonstrates how women’s social, economic and cultural markers can influence the quality of care they receive, including access to pertinent information.

Health information systems are one of the six essential, interconnected building blocks for strengthening health systems established by the World Health Organization (WHO) in 2000. According to the WHO, an effective health information system is ‘one that ensures the production, analysis, dissemination and use of reliable and timely information on health determinants, health systems performance and health status.’^5^

In Peru, women’s clinical information is registered by midwives in their medical history at the local center and on the prenatal control card (The prenatal control card is a cardstock booklet where the midwife records key information about the woman’s pregnancy, including family and personal obstetric history, vaccines, physical and laboratory exam results, blood pressure, pulse, weight and height, pregnancy weight gain curve, fundal height and foetal heartbeat.) that women receive to subsequently bring to their prenatal visits and delivery. Women are responsible for remembering to carry the card, which is critical to ensuring that all their information is recorded and that the current provider has access to it. This is especially important during delivery when the health facility and provider may be different from their prenatal facility and provider. After delivery, women return to their local center without information regarding their delivery process, which is critical to the midwives responsible for postpartum follow-up. There are many points in this prenatal–delivery–postnatal continuum that are prone to errors in recording and subsequent use of information. These errors and potential oversights could compromise both the immediate quality of care and future continuity of care for a woman and her newborn.

With this scenario in mind, our research team implemented an e-Health project to reduce inequities in maternal and infant care in the Peruvian health system by improving the health data and information available at different moments, for different purposes, and at different levels of care. The WawaRed project ‘Connecting for better maternal and child health in Peru’, initially established by the Inter-American Development Bank (IDB), was the focus of implementation research funded by the International Development Research Center (IDRC) of Canada (2013–17).

Learning more about pregnant women’s perspectives regarding their access to information about their own health is crucial to ensuring the systems developed do not violate privacy and autonomy rights or exacerbate existing inequalities related to information access. This is particularly important because there are many inequities related to access to information in populations that receive health services.^6^ Therefore, as part of the WawaRed project, we explored the type of information that pregnant women currently access and their preferences regarding the type of information they want to receive from health providers and other sources.

### The WawaRed project

The WawaRed project includes two main components: (i) an electronic health record (EHR) for prenatal care maintained at the local health center and (ii) text messages (SMS) sent to pregnant women’s cell phones.

The EHR component simplifies and standardizes information collection and reporting and guides health providers in improving their care. Information in the EHR is collected from women by midwives during prenatal care visits. Prior to the introduction of the EHR, midwives had to record information on 10 different formats that fed into various information systems. This non-digitized, poorly standardized process monopolized most of the midwives’ time with patients and contributed to significant data entry errors and delays in reporting data to the regional and national levels.^7,8^ The WawaRed EHR allows midwives to enter the information more quickly, enabling them provide more extensive, in-depth counseling to pregnant women. The EHR also guides health providers through alerts that appear automatically in the system when a woman is missing services or tests or may have abnormal or borderline results.

With an EHR in place and being used consistently by midwives and other primary health providers, the project team worked with the MoH to integrate different previously fragmented maternal health information systems, which helped guarantee the quality of the information at different levels of health policy decision making and the health system.

The text messages component sends general and personalized SMS’s to registered pregnant women free of charge, including reminders about prenatal check-ups and other messages relevant to her gestational age. The general messages provide advice about nutrition, diet and pregnancy-related warning signs (e.g. hypertension), as well as motivational messages with emotional support relevant to their gestational age.^8,9^

## Methods

### Context

This project took place in 2015 in Peru. WawaRed was implemented in the Lima-Callao capital metropolitan area and specifically in Ventanilla, a peri-urban area with 385 000 inhabitants.^10^ Ventanilla residents come from different Andean and Amazon regions of Peru, where oral culture often predominates, and Spanish may not be their mother tongue.

In Ventanilla, the penetration rate of mobile phones is high, with 94% of homes reporting at least one. Mobile phones tend to be shared. Internet use has also grown and stands at 65% of the population age 6 and older. Internet access generally takes place in private homes, on mobile phones and in a mix of places depending on what’s available at a given time.^11^

In Ventanilla, most people receive health care through the MOH. We included participants from all MOH establishments in Ventanilla, including 15 local health centers and one hospital.

### Data collection

This mixed methods study took place during June and July 2014.

For the qualitative component, we held four focus groups with pregnant women in four health centers with the highest numbers of pregnant women. We used a structured guide to explore general and facility-based pregnancy-related information. For general information, we asked about: topics of interest; topics they learned about recently; ways they currently receive information; ways they would like to receive information; and types of information that are useful and interesting. For information from health facilities, we asked about: the content, format and updating of bulletin boards; the content, quality and utility of the information provided in informational talks, prenatal check-ups and individual prenatal control cards; and any suggestions participants had for improvement. An anthropologist with significant experience in qualitative research on gender and sexual and reproductive health conducted the focus groups, which lasted 45–60 min and were audio-recorded.

For the quantitative component, we surveyed pregnant women at all 15 local health centers. Surveys examined Internet use, access to pregnancy-related information in general and at health facilities, and preferences regarding pregnancy-related information. A professional with significant experience carrying out surveys interviewed each pregnant woman in Spanish and recorded her responses on paper-based surveys.

### Data analysis

Focus group recordings were transcribed into text in Spanish. First, we read all the transcripts to identify initial themes and develop an initial codebook. Next, we applied the initial codebook to one focus group to test the codes and finalize the codebook. Then, we applied the codebook to the remaining transcripts. Finally, we analyzed the quotes for each code to identify main messages and similarities and differences across the focus groups.

For the quantitative component, we carried out descriptive data analysis including frequencies and percentages to describe the results. The data were analyzed in Stata version 12.0.

### Ethics

This study was approved by the Institutional Review Board of the Universidad Peruana Cayetano Heredia and approval was in place prior to study initiation.

## Results

Thirty-four pregnant women from Ventanilla who were 18–42 years of age participated in the focus groups. Details about participants are provided in Table 1.

**Table 1:** Focus group participants’ characteristics

Focus Group number	Health facility	Number of participants	Average age of participants	Age range of participants
1	Ciudad Pachacutec Health Center	10	27	20–35
2	Ciudad Pachacutec Health Center	7	26	18–40
3	Mi Peru Health Center	11	24	18–32
4	Ventanilla Hospital	6	32	20–42
Total		34	27	18–42

Four hundred and three pregnant women from Ventanilla participated in the survey. Their mean age was 26.0 (standard deviation 6.6), 232 (57.6%) had secondary education and 156 (38.7%) were pregnant for the first time.

Results are organized by theme and sub-theme and supported by participant quotes and survey information. Table 2 summarizes the link between the (i) study objectives, (ii) topics explored, (iii) themes and (iv) sub-themes.

**Table 2:** Specific objectives, topics explored and resulting overall themes and sub-themes

Specific objective	Topic explored during focus group	Resulting overall theme	Resulting sub-themes
To explore what kind of information pregnant women need and where they look for it	Information that pregnant women believe they need	Type of information will depend on the pregnant woman’s factors or characteristics (number of previous pregnancies, pregnant woman’s age)	Care-related activities to be able to have a healthy pregnancy and baby
			Healthy diet and nutrition
			Pregnancy-related warning signs
			Visits in the health center
			Procedures that must be done to attend their delivery
	Source where pregnant women obtain information	The pregnant woman’s environment has an influence	Familiar surroundings
			Community (neighbors)
			Mothers and mothers-in-law
			Other pregnant women
			Internet
			Health facilities
To explore the information that pregnant women receive at the health facility	Quality of the information that pregnant women received at the health facility	The majority of pregnant women’s doubts are resolved by health personnel	Very technical language that sometimes is not understood
		Depends on the attitude, availability and language of the health personnel	Health staff are not patient
			Sometimes pregnant women do not feel comfortable asking questions
			Information is primarily given at the initial prenatal check-ups
	Forms of communication used by the health facility to give information to pregnant women	Through bulletin boards and during informational talks and prenatal check-ups	Information on bulletin boards is useful and brings new knowledge
			Information on bulletin boards is not updated frequently and is often boring and text-heavy
			The most pleasant bulletin boards are those that are clean and have images, less text, large letters and a variety of colors
			The informational talks are rewarding because pregnant women can interact and share experiences with other pregnant women
			During the informational talks the health personnel make use of different materials and methods, which makes the learning more didactic
			The information provided during the first prenatal check-up is appropriate
			Pregnant women would like to receive information through videos while waiting to be seen at the health facility
	Other ways of communicating with pregnant women	Communication mechanisms that allows them to stay at home	Internet
			Radio
			Television programs or videos

### The information that pregnant women need depends on their socio-demographic characteristics and factors associated with their pregnancy

Most pregnant women agree that the information they require depends on different factors or circumstances. It may depend on their gestational stage and whether it is their first pregnancy, since women who are pregnant for the first time—the so-called ‘first-timers’—have the greatest uncertainties. Participants proposed that information be differentiated according to how each woman’s pregnancy progresses and what number pregnancy it is.

‘First-timers’ said that from the moment they found out about their pregnancy, they required diverse information, including the birth process, despite being at the beginning of their pregnancy. Those who were not ‘first-timers’ also wanted information. They pointed out that in different pregnancies, their symptoms are not necessarily the same, which generates new uncertainties and information needs.

They [Pregnancies] are different… My personal experience is that with the second baby, my symptoms were similar (to my first pregnancy), my births (as well), but now this [my third pregnancy] is different. I have had other discomforts, other symptoms… other pains. (Focus Group 1)

The woman’s age is also an important factor in differentiating information needs. For example, younger women are interested in food, vaccines and permitted medications while older women are more interested in risks that could arise during pregnancy and whether their child is developing properly and will be born healthy.

In my first pregnancy, I did not worry much because I was young, and when you’re young you do not care about anything (…) but when (a woman’s) age advances, that’s where the problems come from. There you have to take care of yourself. (Focus Group 2)

### Pregnant women’s social networks influence their sources of information

Most participants sought to clarify their doubts through various sources. Sources included people in their immediate environment such as mothers, mothers-in-law, neighbors or other pregnant women.

Other sources were Internet websites, which the women—especially the youngest participants—consulted regularly, even daily. One participant even identified an international virtual forum specialized in pregnancy and parenting, where she shared experiences with other pregnant women and could resolve most of her doubts.

[BabyCenter]… is well-known… And there the information is sent according to (your) due date… When one enters, there is a forum of first-time pregnant mothers. I am in the first-time (forum) and they send me information. I also ask (questions) there, and we always exchange ideas, we exchange information. (Focus Group 4).

On the survey, 47.4% of pregnant women reported using the Internet in the last month. The main reason for Internet use was to access social networks (63.9%) or search for different types of information (62.3%).

Many participants, especially those who had attended informational talks at the health facility, preferred these talks to receive information since they can discuss shared experiences and doubts with other women. They reported that sharing with their peers is easier since communication is more fluid and they feel less shy and more confident expressing their doubts.

In the survey, most pregnant women reported that they share information with other pregnant women (93.4%). This usually happens when they are in the waiting room for their prenatal check-ups (89.4%).

### The information received during prenatal check-ups resolved many but not all of women’s doubts

When consulted about the information they received during their prenatal check-ups at the health facility, most women were satisfied. They reported that in many cases, their doubts were resolved by the health professionals:

It [The information] depends on the patient’s concern… We ask about them [our concerns] and they [the health personnel] answer. (Focus Group 1).

Many participants mentioned that sometimes their information needs were not completely met during their prenatal check-ups. They reported that they received a lot of information during their initial check-ups, but that the information lessened as they progressed in their pregnancies, since the check-ups focused more on medical issues.

When you come to your first appointment [prenatal check-up], they [the health personnel] explain everything… but afterwards, they do not explain (things) to you. (Focus Group 2)

### The quality of information received at health facilities depended on health personnel’s attitude and language

Pregnant women valued the pregnancy-related information they received at the health facility since they perceived it to be authorized information. They were especially interested in information related to their diet, since this information is useful in their daily lives. They also indicated that they followed the health personnel’s recommendations.

Good nutrition… my food has changed a lot. Before it [my diet] was messy… Now with everything… that they [the health personnel] tell me… the food, now it’s better already … Yes, it [the information] has affected everything… (Focus Group 4)

One aspect that focus group participants constantly highlighted is the importance they place on the health personnel’s treatment, attitude and language during their prenatal check-ups.

Well, yes, when they [the health personnel] talk to you… (and are) nice, patient… we [clients] come to understand. But sometimes they tell you things in a bad way… (Then), I do not ask anymore. They [the health personnel] get upset. (Focus Group 1)

Interestingly, in the survey, 72.2% of pregnant women reported that they felt that the most trustworthy information came from their midwives, not from their doctors. This may be due to differences in treatment, attitude and languages between the two types of providers.

### Health facilities communicate with pregnant women through various channels

In the survey, 89.9% of pregnant women were satisfied or very satisfied with the information received at health facilities.

#### Bulletin boards

Almost all participants assured that they read the bulletin boards in the health facility where they receive their prenatal care since, generally, they must wait for some amount of time before receiving their care. They affirmed that the information presented is useful since it provides new knowledge.

Participants pointed out that the bulletin boards present information on pregnancy-appropriate diet and exercises, warning signs during pregnancy, vaccines for pregnant women and babies, breastfeeding, newborn care and contraceptive methods.

I think we should highlight all vaccines more, because… mothers, maybe we do not know (about them). I think that on the bulletin board there must be (information about) the timing for each vaccine… what each vaccine is for… that you have to get it. (Focus Group 2)

Some participants mentioned that the bulletin boards are not updated regularly. This means that the time they spend waiting before their multiple prenatal check-ups is not put to optimal use since the information is often the same throughout much of their pregnancy, instead of being modified and updated to promote acquisition of new knowledge. Participants also mentioned that bulletin boards often contain significant amounts of text, instead of a balance of text, images and graphics.

In my health center, there are (bulletin boards), but they have the same posters, (which) do not change… because they [other women] say that in other health centers, they change them [the bulletin boards]… But here… they are the same. (Focus Group 4)

In the survey, almost all pregnant women (85.6%) reported that they always or almost always review these bulletin boards. The great majority also affirmed that the information presented is useful (82.2%), especially if it gives them new knowledge.

#### Informational talks

Only some focus group participants had attended an informational talk at the health facility where they received their prenatal check-ups. Among those who did not participate in these talks, they did not do so because they lacked time due to their daily activities and responsibilities or they did not know about these activities.

Those who attended informational talks or psychoprophylaxis sessions recounted their experiences with great pleasure. They said that these sessions are very useful for acquiring new knowledge and that the information provided is more detailed and in-depth than the information during their prenatal check-ups. They also found sharing with their peers to be very rewarding.

In the talks, we can discuss a topic, and they [the health personnel] provide very good explanations about that topic… We learn specifically about that [topic]… It would be good to explain each month how it [the talk] will be… The questions would be more detailed. (Focus Group 3)

Pregnant women also valued the informational talks because the health personnel make use of different materials and methods, making the learning process more didactic.

Like every mom, it attracts our attention when there are new things… For example, during delivery, they said that they put a pump to quickly reach the placenta, and that causes some pain (…). She [the health worker] also brings videos about… the final stage of delivery and it also shows what the birth, the delivery are like. (Focus Group 1)

According to the surveys, only 9.2% of the pregnant women had attended at least one informational talk. Of these, a very high 91.9% rated the talks as very good or good and 97.3% found them very useful or useful.

### Other ways pregnant women would like to receive information about their pregnancy

Participants mentioned that they would be very interested in receiving reliable pregnancy-related information through sources that are accessible to them at home or during their daily activities.

Internet is one means by which pregnant women would like to receive authorized information from a reliable source, such as health personnel, the health system or another high-quality source. Participants stated that this could be very helpful for them and that it could also their partners’ access to information about their pregnancy. This is important since many pregnant women stated that their partners do not attend their prenatal check-ups due to work obligations.

I cannot keep going (with my) normal (life), because I have to be on medical rest. I still… I would like information to be sent to me over the Internet, right? (Focus Group 3)

One participant mentioned the option of a radio program about pregnancy-related topics:

When you are doing your things, sometimes you do not have time to come here [to the health facility], because of work, any other reason. (Focus Group 1)

Most participants approved of the idea of receiving information from their prenatal control card through the Internet. Even those who were not regular Internet users said that if they were offered virtual access to their prenatal information, they would use the Internet more frequently. Current widespread, low-cost accessibility to the Internet in Peru makes the proposal more attractive to them.

It would be a reason to have an interest in the Internet, because as I say, most of my siblings… are involved in the Internet. (Focus Group 3)

In addition, most participants affirmed that the information on their prenatal cards is very useful.

It was thanks to the prenatal card that I realized that I was not doing well with my weight… according to the stage of pregnancy that I was in… When I asked the doctor, he told me that I had to gain weight. (Focus Group 1)

On the survey, most women (79.4%) affirmed that they would be interested in receiving information from their prenatal control card through the Internet.

## Discussion

### Main findings of this study

Pregnant women in our study described how the information they need during pregnancy depends on individual factors such as their age and number of previous pregnancies and on their social networks and environment. Women looked to people in their immediate environment for pregnancy-related advice and information, but also greatly valued high-quality, timely and targeted information from health personnel and facilities through in-person interactions and via SMS messages received. Participants helped to elucidate how their information needs—which include not only reliable information but also the opportunity for responses to their questions and doubts in a warm, caring environment—can be met both in person in individual and group settings and using information and communication technologies (ICTs), such as SMS to mobile phones or accessing health records via the Internet.

### What is already known on this topic

This study affirmed findings from other contexts, that a warm client-provider care interaction is key for meeting women’s immediate needs, ensuring that women continue to access services and improving health outcomes.^12^ When pregnant women perceive inadequate treatment, inappropriate attitude or complex language by health personnel, this may limit or even block the clients’ communication with personnel. Participants described that when midwives or obstetricians are in a bad mood or being impatient, patients observe this and literally do not dare to ask questions because they are afraid. In turn, clients’ information needs may not be transmitted effectively or at all and therefore not adequately met. By contrast, when pregnant women perceive warmth in the care interaction, this increases clients’ confidence in and comfort with health personnel and encourages clients to express their doubts and needs. The importance of health providers being good communicators and giving clear messages has been demonstrated in previous studies and affirms the importance that pregnant women place on receiving information from health care providers that they can understand and use.^13^

### What this study adds

Our study results demonstrated the importance of providing additional information to pregnant women in health facilities and using ICTs. If used effectively with other in-person communications media, the resulting quality and uptake of information to pregnant women can be increased significantly.

When engaged about communication between health care services and pregnant women, study participants preferred informational talks and valued bulletin boards. Women preferred informational talks since they combine three elements that are important to them: access to high-quality information in a dynamic, interactive context; the opportunity to ask skilled providers about their questions and doubts; and a space where they can share experiences with other pregnant women.

Women also found that bulletin boards were useful for accessing information since they are always available. However, participants noted that many bulletin boards are not updated frequently or do not present the information in a population-friendly way. The presentation of dense written information could exacerbate inequalities between women from different cultural backgrounds such as those living in Ventanilla.

For the reasons explained above, and as part of the WawaRed project, we trained 70 midwives in how to: carry out simple yet high-quality analysis of the information registered in the EHR; and present the results on bulletin boards using infographics that are accurate, informative, population-appropriate, understandable and attractive. To encourage frequent updating of bulletin boards in the health facilities participating in the project, we worked with regional health authorities to organize a ‘best bulletin board’ contest.

When engaged about communication between health care services and pregnant women, participants affirmed their interest in receiving information through ICTs such as SMS and online provision of the EHR. Other studies have shown that pregnant women are interested in receiving pregnancy-related information through SMS.^14^ Women’s interest in technologies motivated us, as part of the WawaRed project, to continue sending SMS messages with pregnancy-related information to pregnant women’s cell phones throughout the entire pregnancy, using easy-to-understand, friendly language. An important advantage of SMS is that it is accessible even with the simplest, lowest-cost cellular phone, at no cost to the recipient.

Provision of online access to a pregnant woman’s EHR is another key use of ICTs during pregnancy. Online access is important for increasing each woman’s access to her own health information. Having ongoing access to their medical information can give patients, and women in particular, the resources to request additional treatment and care or more detailed explanations from their providers. This can be empowering for female patients who may have hierarchical relationships with the health system. Online access could also allow the pregnant woman to share information with her partner so that he can be more involved in the pregnancy process and learn about and support the care that she needs at home. There are other studies that show that the active involvement of the partner during pregnancy can improve the child’s development.^15^ Participants here did not voice concerns about their autonomy once their partners are able to access their health information. Despite this, issues of privacy, confidentiality and autonomous decision making should be further explored, to avoid exacerbating gender inequalities.

In 2016, we handed over the WawaRed EHR to the Peruvian MOH so that it could be institutionalized into the national health information system. The WawaRed EHR has now been scaled up to 2240 midwives from 646 health facilities in 18 (72%) of Peru’s 25 regions. The system has already registered more than 50 000 women. It should be noted that at the outset of the project, only 6000 women were expected to benefit.

In conclusion, the type of information that pregnant women want and need depends on their personal characteristics, their past pregnancy experiences and their current questions, doubts and discomforts. Interestingly, participants did not mention other factors that have been shown to impact health equity, including income, location and livelihoods. Women prefer to look for information in their immediate environment and recognize that the Internet is an excellent potential information source, particularly since it can be high-quality information that is accessed at home. The quality of the information that pregnant women receive at health facilities could be improved by including testimonies of local pregnant women.

To improve maternal health, the implementation of stronger health information systems with innovative tools such as EHR can lead to important gains; but this alone is not sufficient. It is also essential to provide individualized health information to pregnant women in an understandable, friendly manner so that they can maximize their understanding of their pregnancy, follow care and other recommendations, and optimize outcomes for themselves and their newborns.

### Limitations of this study

The primary limitations relate to the exploratory nature of the study, and specifically the introduction of e-Health innovations to support women’s pregnancy-related information needs. Given that some of the topics explored were hypothetical, such as personal digital access to the EHR, it may have been difficult for women to fully comprehend how they would interact with this innovation. Additionally, participants may have felt a certain pressure to endorse the innovations being discussed, to please the research team members.
